# Primary myelofibrosis marrow-derived CD14^+^/CD34^-^ monocytes induce myelofibrosis-like phenotype in immunodeficient mice and give rise to megakaryocytes

**DOI:** 10.1371/journal.pone.0222912

**Published:** 2019-09-30

**Authors:** Taghi Manshouri, Srdan Verstovsek, David M. Harris, Ivo Veletic, Xiaorui Zhang, Sean M. Post, Carlos E. Bueso-Ramos, Zeev Estrov

**Affiliations:** 1 Department of Leukemia, The University of Texas MD Anderson Cancer Center, Houston, TX, United States of America; 2 Department of Hematopathology, The University of Texas MD Anderson Cancer Center, Houston, TX, United States of America; European Institute of Oncology, ITALY

## Abstract

To confirm that neoplastic monocyte-derived collagen- and fibronectin-producing fibrocytes induce bone marrow (BM) fibrosis in primary myelofibrosis (PMF), we injected PMF BM-derived fibrocyte-precursor CD14^+^/CD34^-^ monocytes into the tail vein of NOD-SCID-γ (NSG) mice. PMF BM-derived CD14^+^/CD34^-^ monocytes engrafted and induced a PMF-like phenotype with splenomegaly, myeloid hyperplasia with clusters of atypical megakaryocytes, persistence of the *JAK2*^*V617F*^ mutation, and BM and spleen fibrosis. As control we used normal human BM-derived CD14^+^/CD34^-^ monocytes. These monocytes also engrafted and gave rise to normal megakaryocytes that, like PMF CD14^+^/CD34^-^-derived megakaryocytes, expressed HLA-ABC and human CD42b antigens. Using 2 clonogenic assays we confirmed that PMF and normal BM-derived CD14^+^/CD34^-^ monocytes give rise to megakaryocyte colony-forming cells, suggesting that a subpopulation BM monocytes harbors megakaryocyte progenitor capacity. Taken together, our data suggest that PMF monocytes induce myelofibrosis-like phenotype in immunodeficient mice and that PMF and normal BM-derived CD14^+^/CD34^-^ monocytes give rise to megakaryocyte progenitor cells.

## Introduction

Primary myelofibrosis (PMF) is a myeloproliferative neoplasm characterized by progressive bone marrow (BM) fibrosis.[1, 2] In approximately 90% of patients with PMF the Janus kinase pathway is constitutively activated by a mutation either in the Janus kinase-2 (JAK2), calreticulin (CALR), or the thrombopoietin receptor MPL gene.[3] Although the majority of patients treated with the JAK1/2 inhibitor ruxolitinib experience a significant improvement in their quality of life, in most patients ruxolitinib does not inhibit or reverse BM fibrosis, nor does it eradicate the neoplastic clone.[4] Currently, allogeneic hematopoietic cell transplantation remains the only curative therapy however no more than 10% of PMF patients are eligible for this procedure, and in most centers treatment-related mortality is relatively high.[5]

Challenging the dogma that BM fibrosis is induced by mesenchymal stromal cells (MSC) overstimulated by cytokines produced by megakaryocytes and platelets,[6, 7] we found that BM fibrosis in PMF is primarily induced by an expanded population of neoplastic fibrocytes.[8] Fibrocytes are monocyte-derived spindle-shaped fibroblast-like cells that express hematopoietic and MSC surface proteins and, like MSC, fibrocytes produce collagen-I, collagen-III and fibronectin.[8] Although constituting less than one percent of the BM cellularity, fibrocytes are recruited to site of tissue injury where they participate both in tissue repair and remodeling.[9, 10] Like in the BM of patients with PMF, fibrocytes participate in the induction of fibrosis in several tissues including the lung, liver, kidney, heart, eye, and skin.[10]

Because we found that PMF BM low-density cells engrafted in NOD-SCID-γ (NSG) mice and induced a PMF-like phenotype,[8] we wondered whether monocytes, the fibrocyte precursors, might elicit a similar effect. We hereby report that purified PMF BM CD14^+^/CD34^-^ monocytes engrafted in NSG mice and induced BM fibrosis. Similarly, normal BM CD14^+^/CD34^-^ cells engrafted in NSG mice and, like PMF BM CD14^+^/CD34^-^ cells, gave rise to human megakaryocytes both in vivo and in vitro.

## Materials and methods

### Specimen processing

Diagnostic BM specimens were obtained from treatment-naïve PMF patients after an MD Anderson Cancer Center Institutional Review Board (MDACC IRB1) approved written informed consent had been obtained. The patients’ clinical data are provided in S1 Table. As controls, normal BM aspirates were obtained from AllCells (Alameda, CA). Low-density cells were fractionated by Ficoll-Paque (Healthcare Bioscience AB, Uppsala, Sweden) gradient separation as has been described previously,[8] followed by fluorescence-activated cell sorting (FACS) of CD14^+^/CD34^-^ cells using anti-CD14(PerCP-CY5.5) and anti-CD34 (FITC) antibodies.

### Immunophenotype analysis of the CD14^+^/CD34^-^ cell population

The purity of the fractionated CD14^+^/CD34^-^ cell population was assessed by flow cytometry (FCM) using anti-CD14 (PerCP) and anti-CD34 (FITC) antibodies. In addition, PMF patients’ and healthy donors’ BM aspirates were further analyzed using antibodies or following 9 cell surface antigens: CD14 (PerCP-Cy5.5), CD34 (FITC), CD45 (Pacific Orange), CD68 (PE), CD3 (APC), HLA-DR (APC-H7), CD41 (PE/Cy7), CD42b (Alexa Fluor 700), and CD61 (BV421) using the FlowJo software v10.5.3 (TreeStar, Ashland, OR) and R-based pipeline for high-dimensional cytometry datasets, as previously described.[11, 12] Briefly, CD14^+^ cells were gated and the expression of each of the above mentioned surface markers was assessed. The antibodies corresponding isotypes were used as controls. In addition, cell surface marker intensities of 4,000 randomly selected cells were obtained in each sample and mapped to a comparable range using arcsinh transformation with a cofactor of 150. Clusters were computationally defined using the unsupervised self-organizing map (SOM) clustering in the FlowSOM package within the R environment (R Foundation for Statistical Computing, Vienna, Austria). The antibodies and their isotype controls used in our study are listed in S2 Table.

### Engraftment and evaluation of human BM-derived CD14^+^/CD34^-^ cells in NSG mice

A total of 30 NOD *scid* gamma mice (NOD.Cg-*Prkdc*^*scid*^*Il2rg*^*tm1Wjl*^*/SzJ*; Jackson Laboratories, Bar Arbor, ME) were included in this study. All animal studies were carried out in strict accordance to an animal protocol approved by the MD Anderson Cancer Center Instructional Animal Care and Utilization Committee (Protocol Number: 00000787-RN02). The animals were euthanized using carbon dioxide as per our protocol and all efforts were made to minimize suffering. Two million PMF or hematologically normal BM-derived CD14^+^/CD34^-^ cells were injected into the tail vein of 4–6 week old female NOD *scid* gamma mice. Engraftment was assessed after two weeks and then once a month thereafter by FCM of mouse peripheral blood (PB) cells using mouse anti-human leukocyte antigen (HLA)-ABC antibody and by allele-specific suppressive quantitative polymerase chain reaction (qASSPCR) analyses for the quantification of the *JAK2*^*V617F*^ exon 14 mutation.[13] Following euthanasia, femur BM cells were flushed out and analyzed by FCM using human-specific anti-HLA-ABC (PE), anti-CD45 (PerCP-Cy5.5) and anti-CD68 (FITC) antibodies.

### *In situ* analysis of human BM-derived CD14^+^/CD34^-^ cells in NSG mice

Femur, sternum and spleen biopsy samples were taken after the animals were euthanized (median time following transplantation, 12 weeks). BM and spleen biopsy morphological analyses were performed using hematoxylin and eosin (H&E) and silver staining as previously described.[8] The degree of BM fibrosis was blindly assessed according to the European consensus criteria.[14] Engraftment of human cells was further assessed by chromogenic immunohistochemistry (IHC) using human-specific anti-HLA-ABC, anti-CD42b, anti-CD3, anti-CD19 and anti-CD34 antibodies in avidin-biotin-peroxidase detection system with 3,3′-diaminobenzidine (DAB) substrate and using nuclear fast red counterstain. Then, HLA-ABC-positive cells were further assessed for megakaryocyte markers (CD41, CD42b) and CD14, or fibrocyte markers (CD45, CD68, procollagen-I) by performing multiplexed fluorescence IHC by tyramide signal amplification (TSA) approach. Specifically, mouse BM sections were stained using 2 different panels (S3 Table) and imaged at 40× resolution using Polaris multispectral system (PerkinElmer, Waltham, MA). To ensure optimal signal separation, each fluorophore and tissue autofuorescence were imaged on separate filters and spectrally unmixed as previously described.[15]

### Megakaryocyte colony culture assays

The megakaryocyte colony-forming capacity of PMF (n = 12) and normal BM-derived CD14^+^/CD34^-^ cells (n = 6) was assessed by using two different megakaryocyte colony culture assays. The collagen-based MegaCult-C assay (StemCell Technologies, Vancouver, BC, Canada) was used in accordance with the manufacturer’s instructions with slight modifications. In brief, 40,000 CD14^+^/CD34^-^ cells were cultured in collagen-containing medium in triplicate in the presence of recombinant human thrombopoietin (TPO; 50 ng/mL), interleukin-3 (IL-3; 10 ng/mL) and IL-6 (10 ng/mL). Colony-forming unit-megakaryocyte (CFU-Meg) colonies were fixed after 12 days of culture, stained with anti-CD42b (Vector Blue) antibodies, counterstained with nuclear fast red, and counted using BX43 upright brightfield microscope (Olympus, Tokyo, Japan). A CFU-Meg colony was defined as a cluster of 3 or more nucleated CD42b^+^ cells. In addition, to further assess antigen co-localization, cells were stained with fluorescent actin (Alexa Fluor 488 phalloidin), CD42b (Alexa Fluor 594) and CD14 (Alexa Fluor 647) antibodies with 4′,6-diamidino-2-phenylindole (DAPI) as the nuclear counterstain.

A previously described, slightly modified methylcellulose CFU-Meg colony culture assay was also used.[8] Briefly, 2×10^5^ PMF or normal BM-derived CD14^+^ cells were mixed with 0.8% methylcellulose in Iscove’s modified Dulbecco’s medium (Invitrogen, Grand Island, NY) supplemented with 10% fetal calf serum (Invitrogen) and 10 ng/mL thrombopoietin. Five-hundred mL of the mix was incubated in Petri dishes at 37°C in a humidified atmosphere of 5% CO_2_ in air. CFU-Meg colonies were scored at day 21 of culture using the Diaphot ELWD 0.3 inverted phase-contrast microscope (Nikon, Tokyo, Japan). A CFU-Meg colony was defined as a cluster of more than 10 large translucent cells or adjacent clusters or 3 or more translucent cells. Single colonies were microaspirated, cytospun onto glass slides and analyzed after May-Grünwald-Giemsa (MGG) staining and immunolabeling with anti-CD42b (DAB) antibodies as described above. To assess differences between colony numbers, the Prism v7.03 software (GraphPad, San Diego CA) was used. Groups were compared using Student’s *t*-test and *P* < 0.05 was considered statistically significant. Fluorescence images were acquired using the Andor Revolution XDi WD spinning disk confocal system with Zyla 4.2 sCMOS camera (Andor Technology, Belfast, UK) and UPlanSApo 30XS silicone oil-immersion objective lens (Olympus, Tokyo, Japan), and adjusted for visual presentation using Imaris v9.1.0 software (Bitplane, Zurich, Switzerland). Antibodies and detection reagents used in the cellular imaging are listed in S4 Table.

## Results and discussion

**Fig 1:**
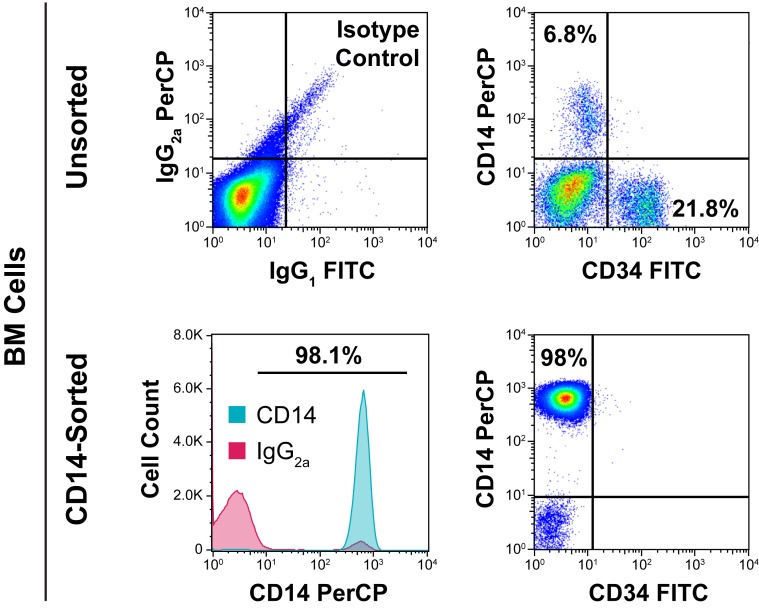
Fractionation and characterization of CD14^+^/CD34^-^ monocytes. Flow cytometry (FCM) analysis of unsorted PMF and normal donor BM-derived low-density cells using anti-immunoglobulin G_2a_ (IgG_2a_) and anti-IgG_1_ isotype control antibodies (left upper panel), and anti-CD14/anti-CD34 monoclonal antibodies (right upper panel) shows that PMF BM CD14^+^ cells do not express the CD34 antigen. In addition, FCM analysis of FACS-sorted PMF BM-derived cells showed that 98.1% of the sorted cells were CD14-positive (left lower panel) and that the sorted CD14^+^ cells were CD34^-^ (right lower panel). Representative single-sample data from experiments performed using 3 different PMF and 2 normal BM cells are depicted.

We have recently found that PMF BM-derived neoplastic fibrocytes induced BM fibrosis. Furthermore, we demonstrated that PMF BM low-density cells engrafted in NSG mice and induced BM and spleen fibrosis.[8] Because fibrocytes are monocyte derived, we wondered whether PMF BM monocytes, would induce BM fibrosis in NSG mice. Using FCM we analyzed PMF and normal donor BM low-density cells and found that CD14^+^ cells do not express the CD34 antigen (Fig 1). Then, using FACS sorting we isolated CD14^+^ BM monocytes from PMF patients’ BM aspirates (n = 3) and, as control, from BM aspirates of healthy individuals (n = 2). Of the CD14^+^ BM cells, 97.6 ± 1.0% were CD14^+^/CD34^-^ (Fig 1), indicating that the FACS-sorted CD14^+^ cell population was devoid of CD34^+^ progenitor cells [16] (Fig 1; right lower panel).

**Fig 2:**
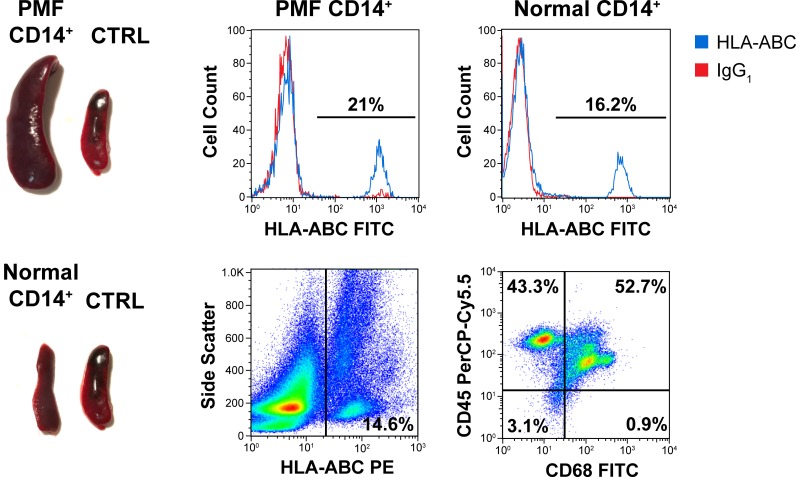
CD14^+^/CD34^-^ monocytes engraft in NSG mice and PMF, but not normal, BM-derived monocytes induce a PMF-like phenotype. Two months after transplantation, 3 mice injected with PMF BM-derived monocytes and 1 mouse injected with normal BM-derived monocytes were euthanized. Shown is a typical large spleen found in all mice transplanted with PMF BM-derived CD14^+^/CD34^-^ monocytes at 1, 2, 3, 4, and 5 months after transplantation but not in mice transplanted with normal BM-derived CD14^+^/CD34^-^ cells or untransplanted mice (left panel). CTRL, control untransplanted mice. HLA-ABC^+^ cells detected in the PB of all transplanted mice (middle and right upper panel), and in the BM of mice transplanted with PMF CD14^+^/CD34^-^ cells (left lower panel), 50% of which co-expressed human CD45 and human CD68 (right lower panel) are shown.

To determine whether PMF patients’ CD14+ cells induce BM fibrosis in NSG mice, we isolated CD14^+^/CD34^-^ BM-derived monocytes from 3 PMF patients and 5 healthy donors and injected the PMF-derived monocytes into 15 NSG mice and normal CD14^+^/CD34^-^ monocytes into 5 NSG mice using the protocol of our previously described xenograft mouse model.[8] Ten uninjected mice were used as control. After two weeks and every 4 weeks thereafter, the mice were bled, and their PB cells were assessed for the presence of HLA-ABC antigen by using flow cytometry. HLA-ABC^+^ cells were detected in the PB of all transplanted mice, but not in control untransplanted mice, until they were euthanized. Two months after transplantation, 3 mice transplanted with PMF CD14^+^/CD34^-^ cells and one mouse transplanted with normal BM CD14^+^/CD34^-^ cells were euthanized. Whereas PB HLA-ABC^+^ cells were detected in all animals of both groups (Fig 2, middle and right upper panels), enlarged spleens were found in PMF but not in normal CD14^+^/CD34^-^-transplanted mice at 1, 2, 3, 4, or 5 month after transplantation (Fig 2; left panel). Remarkably, 14.3% of BM cells obtained from mice transplanted with PMF CD14^+^/CD34^-^ monocytes expressed HLA-ABC and 50% of the HLA-ABC^+^ cells co-expressed human CD45 and human CD68 antigens (Fig 2; middle and right lower panels). Taken together, these data suggest that, similar to PMF and normal BM low-density cells,[8] BM-derived CD14^+^/CD34^-^ cells engraft in NSG mice.

**Fig 3:**
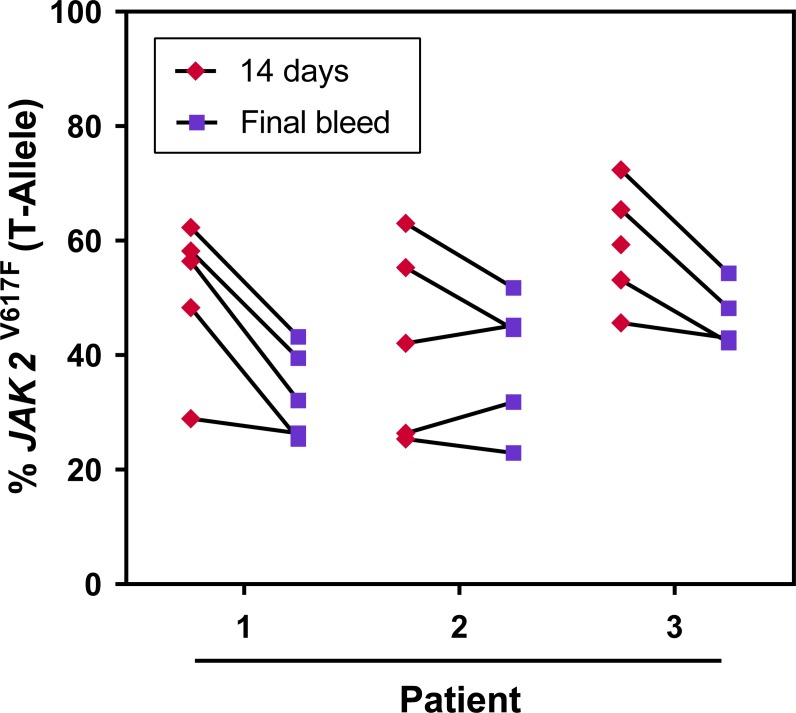
High mutant *JAK2*^V617F^ allele burden is detected in NSG mice transplanted with PMF-derived CD14^+^ cells. The levels of human *JAK2*^V617F^ allele burden were measured in blood samples of NSG mice transplanted with CD14^+^/CD34^-^ BM cells obtained from PMF patient with a *JAK2*^V617F^ mutation. Depicted are the *JAK2*^V617F^ allele burdens of 15 mice transplanted with CD14^+^/CD34^-^ cells from 3 different PMF patients. The animals’ blood was obtained 2 weeks after transplantation and before the animals were euthanized (final bleed). Although there is apparent decline in the *JAK2*^V617F^ allele burden at the time of final bleed, the human mutant *JAK2* variant was still detected at a significant measurable level several months after transplantation.

To confirm that engraftment of human neoplastic BM monocytes persisted, we systematically assessed the level of patient-derived *JAK2*^*V617F*^ mutated neoplastic cells in the BM of mice transplanted with PMF monocytes. Using qASSPCR, we detected in BM cells of mice euthanized at 30, 60, 90, 120, and 150 days after transplantation a *JAK2*^*V617F*^ allele burden of 22.1 ± 19%, 37.5 ± 8%, 26.3 ± 18%, 42.2 ± 10%, and 39.5 ± 2%, respectively. To further determine that engraftment of the PMF CD14+ cell-derived clone persists in the transplanted mice, we measured the levels of human *JAK2*^V617F^ allele burden in the blood of NSG mice transplanted with JAK2-positive CD14^+^/CD34^-^ cells 2 weeks after transplantation and before the animals were euthanized (final bleed). Although we observed a decline in the *JAK2*^V617F^ allele burden at the time of final bleed, the human mutant *JAK2* variant was still detectable at a significant level several months after transplantation (Fig 3).

**Fig 4:**
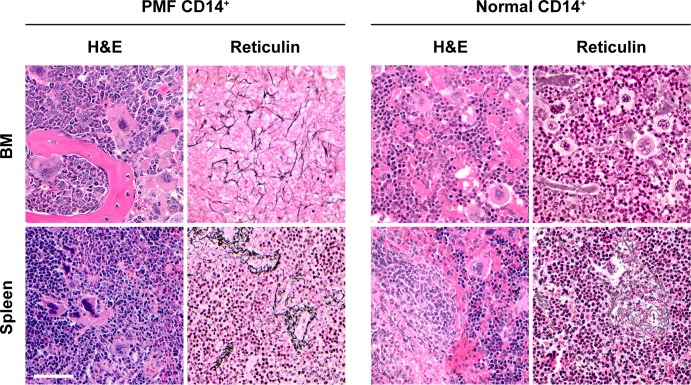
BM and spleen fibrosis is readily detected in NSG mice transplanted with PMF, but not normal, CD14^+^/CD34^-^ BM cells. H&E and reticulin staining of BM (femur or sternum) and spleen sections of mice injected with PMF (left panel) or normal BM CD14^+^/CD34^-^ cells (right panel) are depicted. Notable features in PMF BM-transplanted mice include atypical megakaryopoiesis, anisocytosis, abnormal large nuclear/cytoplasmic ratio, hyperchromatic nuclei and plump lobulation of the nuclei. Reticulin-stained BM and spleen sections from mice injected with PMF BM-derived CD14^+^/CD34^-^ cells (left panel) show increased reticulin fibrosis. In contrast, in mice injected with normal BM-derived CD14^+^/CD34^-^ cells (right panel) hematopoiesis is unaltered, splenic architecture is preserved, and no significant reticulin fiber deposition is observed. Biopsies were taken after the animals were euthanized (median time following transplantation, 12 weeks). Bar, 50 μm.

**Fig 5:**
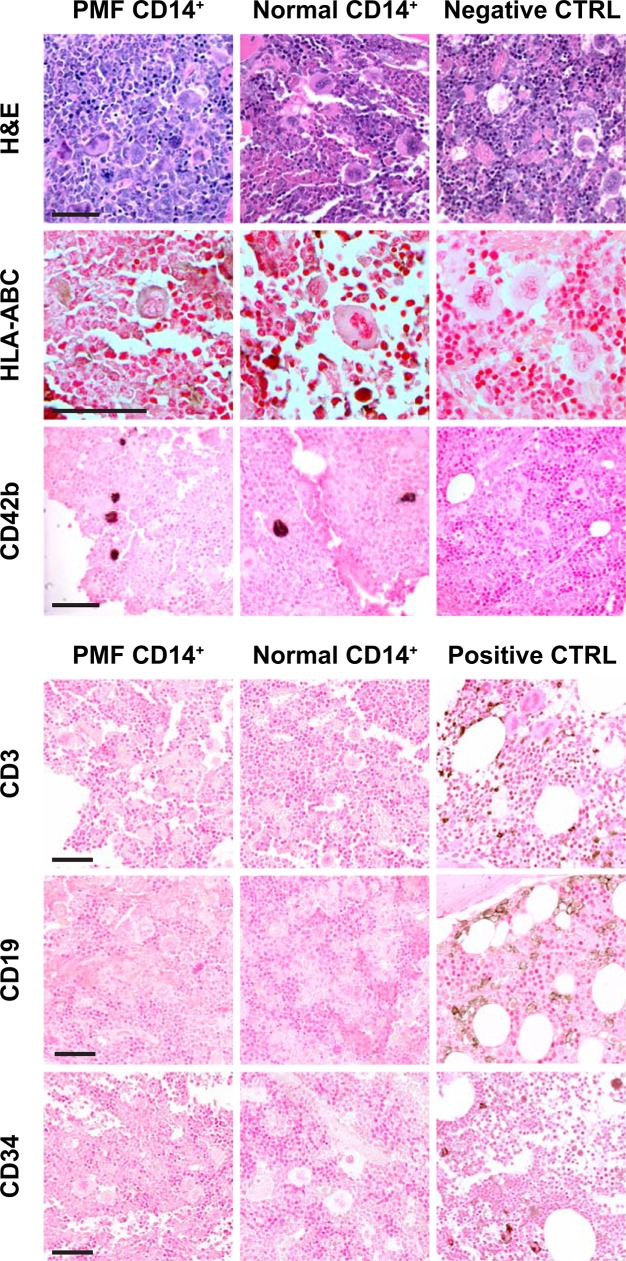
Megakaryocytes in the BM of mice transplanted with human BM CD14^+^/CD34^-^ cells are human-derived. Representative images of mouse femur or sternum BM sections are depicted. Human leukocyte antigen (HLA)-ABC- and human CD42b-positive megakaryocytes (upper panel), but no human CD3^+^, CD19^+^ or CD34^+^ cells (lower panel), were detected by immunohistochemistry in the BM of mice transplanted with either PMF BM-derived CD14^+^/CD34^-^ monocytes (left panel) or normal BM-derived CD14^+^/CD34^-^ monocytes (middle panel). HLA-ABC^+^ or human CD42b^+^ megakaryocytes were not detected in the BM of untransplanted mice (Negative CTRL; right upper panel), whereas CD3^+^, CD19^+^ and CD34^+^ cells were detected in the normal human BM (Positive CTRL; right lower panel). Bars, 50 μm.

**Fig 6:**
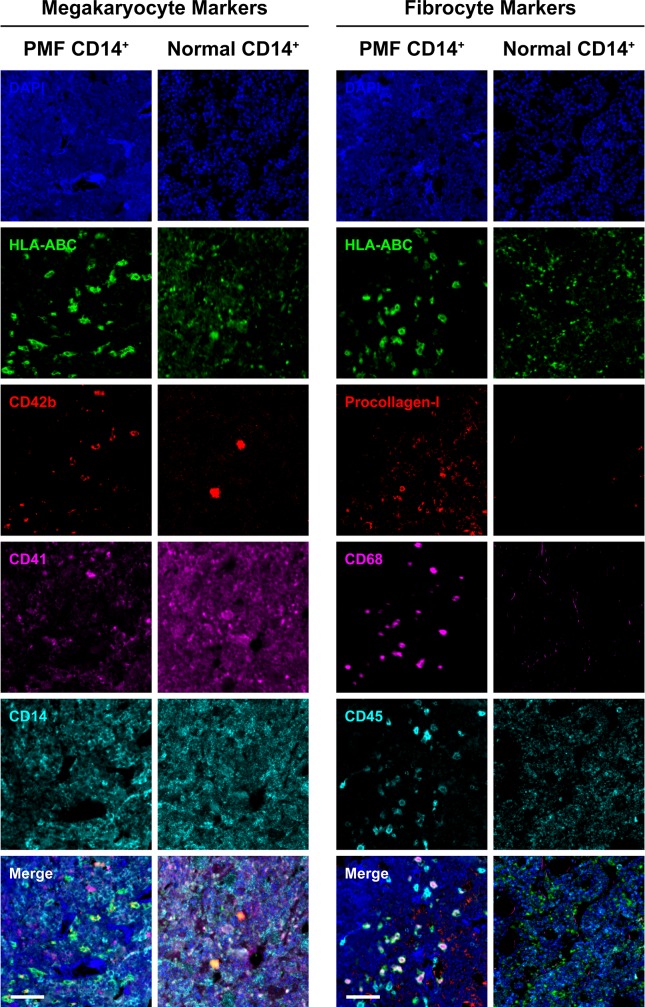
PMF and normal BM-derived CD14^+^ cells transplanted into NSG mice give rise to human megakaryocytes, whereas PMF, but not normal BM CD14^+^ cells give rise to human fibrocytes. Representative images of HLA-ABC^+^ cells in the BM of mice following injection of PMF (n = 3) and normal human BM CD14^+^ cells (n = 2) are depicted. Expression of megakaryocyte (CD41, CD42b; left panel), and fibrocyte markers (CD45, CD68, procollagen-I; right panel) was assessed using multiplexed fluorescence IHC. Nuclei were counterstained using DAPI. Multiple large HLA-ABC^+^/CD41^+^/CD42b^+^ cells corresponding to human megakaryocytes, are observed in the BM of mice injected with PMF or normal CD14^+^ cells (left lower panel; Merge). In contrast, spindle-shaped HLA-ABC^+^/CD45^+^/CD68^+^/procollagen-I^+^ cells, corresponding to human fibrocytes, are observed in the BM of mice injected with PMF patients’ but not normal BM-derived CD14^+^ cells (right lower panel; Merge). Due to cross-reactivity of the CD41 antibodies with mouse cells some HLA-ABC^-^ cells appear to express CD41. No gamma correction was applied. Bars, 50 μm.

Like in patients with PMF and NSG mice transplanted with PMF BM low-density cells,[8] clusters of atypical megakaryocytes with abnormal large nuclear/cytoplasmic ratio, hyperchromatic nuclei with plump nuclear lobulation were identified in the BM and spleen of NSG mice that were transplanted with PMF BM-derived CD14^+^/CD34^-^ monocytes (Fig 4). To assess whether those atypical megakaryocytes were human- or mouse-derived, we used immunohistochemistry. Using this staining we detected megakaryocytes expressing HLA-ABC and human CD42b antigens in BM and spleen biopsy specimens of mice that were transplanted with PMF CD14^+^/CD34^-^ monocytes. Similarly, we detected morphologically normal megakaryocytes expressing HLA-ABC and human CD42b antigens in the BM and spleen biopsies of mice that were transplanted with normal BM-derived CD14^+^/CD34^-^ monocytes but not in BM or spleen biopsies of untransplanted mice (Fig 5). Then, to determine whether BM-derived CD14^+^/CD34^-^ cells gave rise to cells of other hematopoietic lineages we used immunohistochemistry. These stainings did not detect cells expressing human CD3, CD19 or CD34 antigens in all BM and spleen sections of mice transplanted with either PMF or normal monocytes (Fig 5). Further analysis confirmed that PMF and normal BM CD14^+^/CD34^-^ cells transplanted into NSG mice, gave rise to human megakaryocytes (Fig 6; left panel)whereas PMF, but not normal BM CD14^+^/ CD34^-^ cells gave rise to human fibrocytes as assessed by fluorescent immunostaining of BM sections of mice injected with PMF (n = 3) or normal BM CD14^+^ cells (n = 2), using megakaryocyte (CD41, CD42b), and fibrocyte (CD45, CD68, procollagen-I) markers (Fig 6; right panel). Taken together, these data suggested that human BM-derived CD14^+^/CD34^-^ monocytes engrafted in NSG mice and gave rise to human megakaryocytes and fibrocytes.

**Fig 7:**
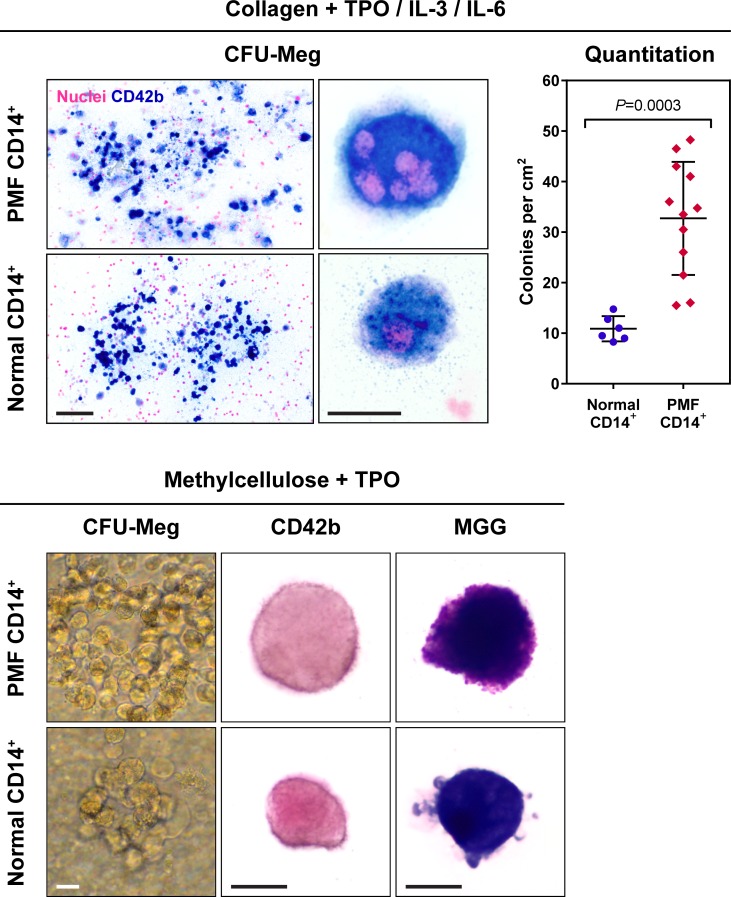
PMF and normal BM monocytes give rise to megakaryocyte colony-forming cells. Depicted are representative images of CFU-Meg colonies grown in collagen medium in the presence of thrombopoietin (TPO), interleukin-3 (IL-3) and IL-6 (upper panel) and CFU-Meg colonies grown in methylcellulose with addition of TPO alone (lower panel). Colonies in the collagen assay were fixed, stained with CD42b antibodies (Vector Blue) and counterstained with nuclear fast red. Megakaryocyte colonies were defined as clusters of 3 or more nucleated CD42b^+^ cells (left upper panel). Enlarged images of the CFU-Meg colonies identified mature megakaryocytes with multi-lobulated nuclei and high cytoplasm-to-nucleus ratio (middle upper panel). Cultures were scored based on total number of colonies counted inside the slide area. As shown (right upper panel), PMF CD14^+^ cells gave rise to a higher number of CFU-Meg colonies than normal CD14^+^ BM cells. Lines with error bars represent mean and standard deviation. Single colonies grown in the methylcellulose culture assay (left lower panel) were microaspirated, cytospun and stained with May-Grünwald-Giemsa (MGG) (right lower panel), and immunostained with anti-CD42b antibodies (middle lower panel), showing that the colonies consisted of micro-megakaryocytes that express CD42b cell surface antigen, typically detected in megakaryocytes. Bars, 100 μm (upper left panel), 20 μm (middle upper panel and lower panels).

**Fig 8:**
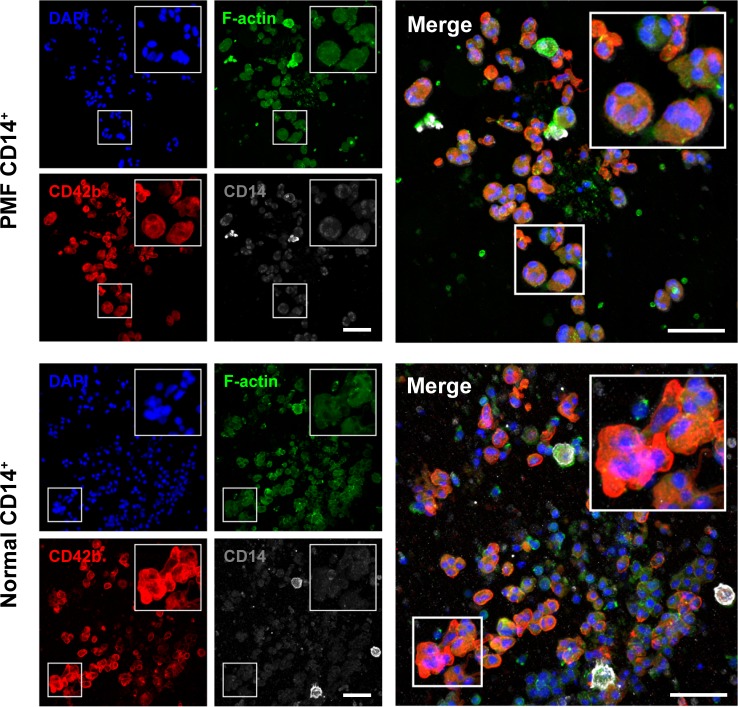
Immunofluorescence staining of the CFU-Meg colonies grown in collagen culture medium. Representative confocal images of single CFU-Meg colonies fluorescently stained with F-actin (Alexa Fluor 488 phalloidin), CD42b (Alexa Fluor 594) and CD14(Alexa Fluor 647) antibodies and nuclear DAPI stain. Depicted are CFU-Meg colony-derived multi-lobulated megakaryocytes from PMF and normal BM monocytes. Z-stacks were acquired at Nyquist sampling frequency with a 0.44-μm step size. No gamma adjustment was applied. Bars, 50 μm.

**Fig 9:**
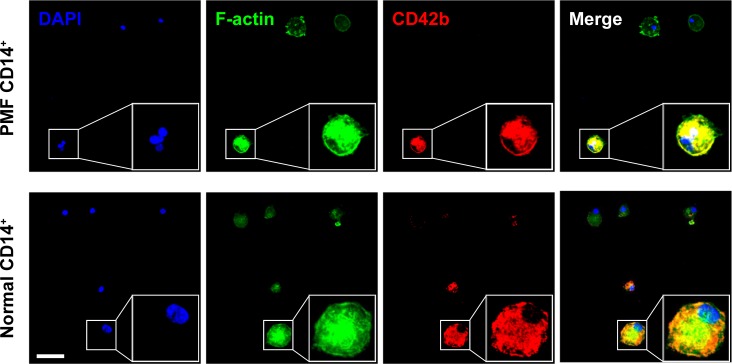
Immunofluorescent analysis of microaspirated PMF and normal BM CD14^+^/CD34^-^ cell-derived megakaryocyte colonies. CFU-Meg colonies were cultured in methylcellulose culture medium supplemented with thrombopoietin (TPO). Cells from microaspirated cytospun colonies were fluorescently stained using F-actin (Alexa Fluor 488 phalloidin) and CD42b (Alexa Fluor 594) antibodies. Multiple CD42b^+^ cells with or without nuclear lobulation corresponding to promegakaryocytes and micro-megakaryocytes, respectively. DAPI was used as the nuclear counterstain. No gamma adjustment was applied. Bars, 50 μm.

To further confirm these findings we cultured 12 PMF and 6 normal BM-derived CD14^+^/CD34^-^ cells in 2 different megakaryocyte colony culture assays. After 3 weeks in culture, PMF BM CD14^+^/CD34^-^ cells gave rise to 25–35 megakaryocyte colonies whereas normal BM CD14^+^/CD34^-^ cells gave rise to 10–15 colonies. Similarly, in the collagen culture assay PMF BM CD14^+^/CD34^-^ gave rise to a larger number of colonies than normal BM CD14^+^/CD34^-^ cells (Fig 7, right upper panel). Immunocytochemistry (Fig 7) and fluorescent immunostaining of colonies grown in the CFU-Meg collagen colony culture assay (Fig 8), as well as cytospun single microaspirated colonies grown in the methylcellulose culture assay (Fig 9), confirmed that the colonies consisted of megakaryocytes with lobulated nuclei or micro-megakaryocytes expressing CD42b, suggesting that a fraction of CD14^+^ BM monocytes harbor megakaryocyte colony-forming cell capacity.

**Fig 10:**
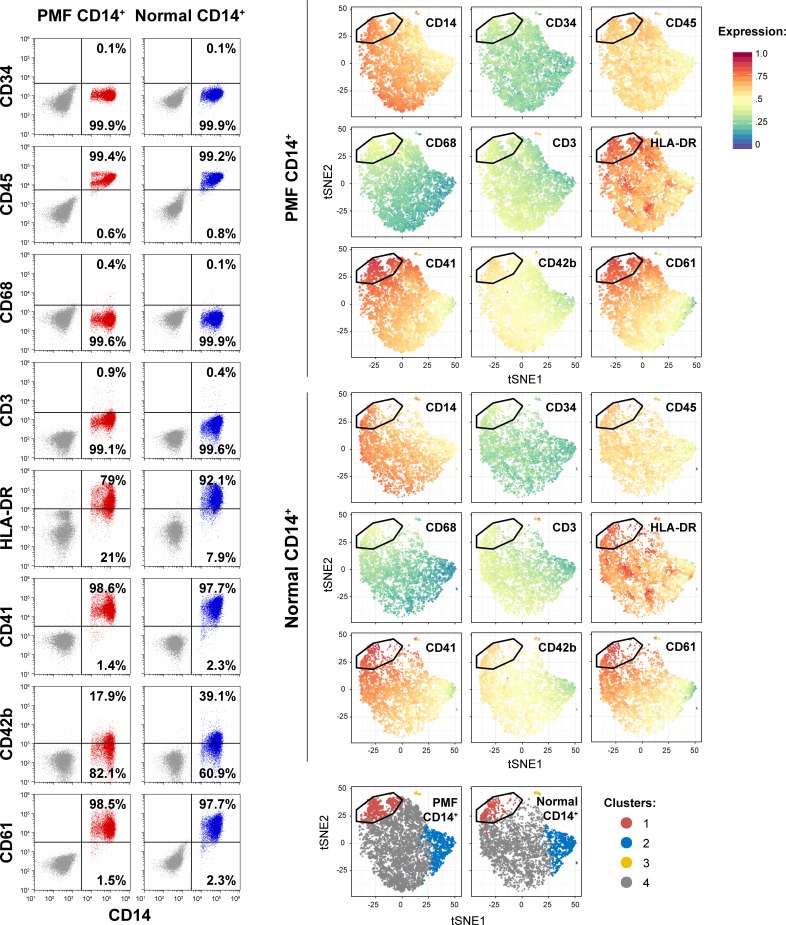
Immunophenotype of PMF and normal BM-derived CD14^+^ cells. PMF (n = 5) and normal BM-derived CD14^+^ cells (n = 3) were analyzed by flow cytometry using 9 lineage markers. The dot plots depict representative analyses of the 9 surface markers detected on CD14^+^ cells (left panel), and the t-stochastic neighbor embedding (t-SNE) plots depict the cell-cell similarity based on the expression of all 9 markers (right upper panel) and corresponding cluster annotation (right lower panel). In addition to CD14 and CD34, expression profiles of CD45 (pan-leukocyte marker), CD68 (macrophage marker), CD3 (T-cell marker), HLA-DR (antigen-presenting cell marker) and CD41, CD42b and CD61 (megakaryocyte markers) are shown. In dot plots (left panel), red dots represent PMF cells, blue dots represent normal cells, and grey dots represent cells stained with the corresponding antibody’s isotype control. Percentages of positive and negative populations of each marker were calculated using the depicted gates. In t-SNE plots (right panel), dots represent random 2,000 individual cells from each analyzed sample. For the purpose of clustering, all marker expressions were transformed using arcsinh (inverse hyperbolic sine) with a cofactor of 150. For visualization in t-SNE plots, expressions were further scaled to values between 0 and 1 using low (1%) and high (99%) percentiles as the boundary. The depicted gates represent computed boundaries of a cluster corresponding to megakaryocyte progenitors (cluster 1).

Because we found that a subpopulation of CD14^+^ monocytes harbors megakaryocyte progenitor capacity, we wondered whether a subset(s) of CD14^+^ monocytes expresses megakaryocyte lineage surface markers. To answer this question we performed a high-parametric immunophenotype analysis of PMF and normal BM CD14^+^ cells. We found that the vast majority of CD14^+^ cells co-express CD45 and HLA-DR, but not CD34, CD68 or CD3 antigens (Fig 10; left panel) and in agreement with previous reports,[12, 17–22] we found that subpopulations of PMF and normal CD14^+^ BM cells express the cell surface antigens CD41 and CD61, commonly detected on immature megakaryocytes, and CD42b, commonly detected on mature megakaryocytes. By performing unsupervised cluster analysis using all 9 markers, we identified a distinct subpopulation of CD14^+^ cells (cluster 1) that expresses all 3 megakaryocyte markers (Fig 10; right panel). The CD14^+^ cells that expressed megakaryocyte antigens were small round monocytes as assessed by lateral and forward scatter analysis and by morphological criteria, indicating that subsets of CD14^+^ BM monocytes are primed to differentiate into cells of the megakaryocyte lineage.

Taken together, our data suggest that PMF and normal BM CD14^+^/CD34^-^ monocytes engraft in NSG mice and that PMF BM fibrocyte precursor CD14^+^/CD34^-^ monocytes induce a PMF-like phenotype with splenomegaly and BM fibrosis in NSG mice. In addition, our data suggest that a subpopulation of CD14^+^/CD34^*-*^ monocytes give rise to CFU-Meg but not an upstream progenitor cell.[23] Whether monocyte-derived megakaryocytes play a role in the pathogenesis of PMF remains to be determined.

## Supporting information

S1 Table Patients’ characteristics. (PDF)

S2 Table Flow cytometry antibodies and isotype controls. (PDF)

S3 Table Multiplexed fluorescence immunohistochemistry assays. (PDF)

S4 Table Cellular imaging detection reagents and antibodies. (PDF)
