# Supplementary material for: Primary myelofibrosis marrow-derived CD14+/CD34- monocytes induce myelofibrosis-like phenotype in immunodeficient mice and give rise to megakaryocytes
Source: PLoS One. 2019 Sep 30;14(9):e0222912. doi: 10.1371/journal.pone.0222912 (PMC6768666; doi:10.1371/journal.pone.0222912)
Supplement: S1 Table — (PDF) [file pone.0222912.s001.pdf]

S1 Table. Patients' characteristics.

Patient	1	2	3
Age (years)	73	61	63
Sex	Female	Female	Male
Diagnosis (WHO 2016 criteria)	PMF	PMF	PMF
Time from diagnosis (months)	0.5	2.8	1.0
Chronic phase disease	Yes	Yes	Yes
BM fibrosis grade (EC 2005 criteria)	MF-2	MF-2	MF-2
IPSS risk category	Int-2	Int-2	Low
Leukocyte count ($\times 10^9$/L)	20.1	13.2	13.1
Hemoglobin (g/dL)	13.1	9.8	12.9
Platelet count ($\times 10^9$/L)	44	289	188
Monocytes (% leukocytes)	4.0	4.3	1.0
Circulating blasts (% leukocytes)	1.0	0	0
LDH (U/L)	1147	1062	1444
Constitutional symptoms	No	Yes	No
Hepatosplenomegaly	No	No	No
JAK2 (V617F) allele burden (%)	67.2%	70.0%	85.9%
Karyotype abnormalities	None	None	None
Previous or current treatment	No	No	No

WHO, World Health Organization; PMF, primary myelofibrosis; BM, bone marrow; EC, European consensus; IPSS, International Prognostic Scoring System; LDH, lactate dehydrogenase
