# Supplementary material for: Primary myelofibrosis marrow-derived CD14+/CD34- monocytes induce myelofibrosis-like phenotype in immunodeficient mice and give rise to megakaryocytes
Source: PLoS One. 2019 Sep 30;14(9):e0222912. doi: 10.1371/journal.pone.0222912 (PMC6768666; doi:10.1371/journal.pone.0222912)
Supplement: S2 Table — (PDF) [file pone.0222912.s002.pdf]

S2 Table. Flow cytometry antibodies and isotype controls.

Name (clone)	Fluorophore	Supplier	Catalog no.
**Antibodies**			
CD3 (SK7)	APC	BD Biosciences, San Jose, CA	340440
CD14 (MφP9)	PerCP	BD Biosciences, San Jose, CA	340585
CD14 (MφP9)	PerCP-Cy5.5	BD Biosciences, San Jose, CA	562692
CD34 (8G12)	FITC	BD Biosciences, San Jose, CA	348053
CD41 (HIP8)	PE/Cy7	BioLegend, San Diego, CA	303718
CD42b (HIP1)	Alexa Fluor 700	BioLegend, San Diego, CA	303927
CD45 (HI30)	Pacific Orange	Invitrogen, Waltham, MA	MHCD4530
CD45 (2D1)	PerCP-Cy5.5	BD Biosciences, San Jose, CA	340952
CD61 (VIPL2)	BV421	BD Biosciences, San Jose, CA	744381
CD68 (Y1/82A)	FITC	BD Biosciences, San Jose, CA	562117
CD68 (Y1/82A)	PE	BioLegend, San Diego, CA	333808
HLA-ABC (G46-2.6)	FITC	BD Biosciences, San Jose, CA	555552
HLA-ABC (G46-2.6)	PE	BD Biosciences, San Jose, CA	555553
HLA-DR (L243)	APC-H7	BD Biosciences, San Jose, CA	641393
**Isotype controls**			
IgG_1, κ (MOPC-21)	Alexa Fluor 700	BD Biosciences, San Jose, CA	557882
IgG_1, κ (MOPC-21)	APC	BD Biosciences, San Jose, CA	555751
IgG_1, κ (MOPC-21)	FITC	BD Biosciences, San Jose, CA	555748
IgG_1, κ (MOPC-21)	PE	BD Biosciences, San Jose, CA	559320
IgG_1, κ (MOPC-21)	PE/Cy7	BioLegend, San Diego, CA	400125
IgG_1, κ (X40)	BV421	BD Biosciences, San Jose, CA	562438
IgG_1, κ (X40)	PerCP-Cy5.5	BD Biosciences, San Jose, CA	347212
IgG_1, κ	Pacific Orange	Invitrogen, Waltham, MA	MG130
IgG_{2a}, κ (G155-178)	APC-H7	BD Biosciences, San Jose, CA	560897
IgG_{2b}, κ (27-35)	FITC	BD Biosciences, San Jose, CA	555742
IgG_{2b}, κ (27-35)	PerCP-Cy5.5	BD Biosciences, San Jose, CA	558304
IgG_{2b}, κ (MG2b-57)	PE	BioLegend, San Diego, CA	401208
IgG_{2b}, κ (MPC-11)	PerCP	BioLegend, San Diego, CA	400336

CD, cluster of differentiation; HLA, human leukocyte antigen; IgG, immunoglobulin G
