# Supplementary material for: Primary myelofibrosis marrow-derived CD14+/CD34- monocytes induce myelofibrosis-like phenotype in immunodeficient mice and give rise to megakaryocytes
Source: PLoS One. 2019 Sep 30;14(9):e0222912. doi: 10.1371/journal.pone.0222912 (PMC6768666; doi:10.1371/journal.pone.0222912)
Supplement: S3 Table — (PDF) [file pone.0222912.s003.pdf]

S3 Table. Multiplexed fluorescence immunohistochemistry assays.

Staining Order	Megakaryocyte Markers		Fibrocyte Markers	
	**Target**	**Fluorophore**	**Target**	**Fluorophore**
1	CD41	Opal 520	CD68	Opal 520
2	CD42b	Opal 570	Procollagen type I	Opal 570
3	CD14 (SP192)	Opal 690	CD45	Opal 690
4	HLA-ABC	Opal Polaris 780	HLA-ABC	Opal Polaris 780
5	Nuclei	DAPI	Nuclei	DAPI

CD, cluster of differentiation; HLA, human leukocyte antigen; DAPI, 4’,6-diamidino-2-phenylindole
