# Supplementary material for: Primary myelofibrosis marrow-derived CD14+/CD34- monocytes induce myelofibrosis-like phenotype in immunodeficient mice and give rise to megakaryocytes
Source: PLoS One. 2019 Sep 30;14(9):e0222912. doi: 10.1371/journal.pone.0222912 (PMC6768666; doi:10.1371/journal.pone.0222912)
Supplement: S4 Table — (PDF) [file pone.0222912.s004.pdf]

S4 Table. Cellular imaging detection reagents and antibodies.

Name (clone *or* conjugate)	Supplier	Catalog no.
**Primary antibodies**		
CD3 (MRQ-39)	Cell Marque, Rocklin, CA	103R-94
CD14 (SP192)	Abcam, Cambridge, United Kingdom	ab183322
CD14 (1H5D8)	Abcam, Cambridge, United Kingdom	ab181470
CD19 (MRQ-36)	Cell Marque, Rocklin, CA	119M-14
CD34	Novus Biologicals, Centennial, CO	NBP2-38321
CD41 (M148)	Abcam, Cambridge, United Kingdom	ab11024
CD42b (EPR6995)	Abcam, Cambridge, United Kingdom	ab134087
CD45 (MEM-28)	Abcam, Cambridge, United Kingdom	ab8216
CD68 (KP1)	Abcam, Cambridge, United Kingdom	ab955
HLA-ABC (EMR8-5)	Abcam, Cambridge, United Kingdom	ab70328
Procollagen-I (M-58)	Abcam, Cambridge, United Kingdom	ab64409
**Secondary antibodies**		
Broad spectrum IgG (HRP)	Invitrogen, Waltham, MA	87-8963
Mouse/rabbit IgG (HRP)	Vector Laboratories, Burlingame, CA	PK-6200
Rabbit IgG (AP)	Vector Laboratories, Burlingame, CA	AK-5001
Rabbit IgG (Alexa Fluor 594)	Invitrogen, Waltham, MA	A21207
Mouse IgG (Alexa Fluor 647)	Invitrogen, Waltham, MA	A31571
**Fluorescent detection reagents**		
Phalloidin (Alexa Fluor 488)	Invitrogen, Waltham, MA	A12379
DAPI	Invitrogen, Waltham, MA	D3571
Opal 520	PerkinElmer, Waltham, MA	FP1487001KT
Opal 570	PerkinElmer, Waltham, MA	FP1488001KT
Opal 690	PerkinElmer, Waltham, MA	FP1497001KT
Opal Polaris 780	PerkinElmer, Waltham, MA	FP1501001KT
**Chromogenic detection reagents**		
DAB	Vector Laboratories, Burlingame, CA	SK-4100
Vector Blue	Vector Laboratories, Burlingame, CA	SK-5300
Shandon Wright-Giemsa	Thermo Scientific, Waltham, MA	99-907-10
Nuclear fast red	Ricca Chemical, Arlington, TX	R5463200-500A

CD, cluster of differentiation; HLA, human leukocyte antigen; IgG, immunoglobulin G; HRP, horseradish peroxidase; AP, alkaline phosphatase; DAPI, 4’,6-diamidino-2-phenylindole; DAB, 3,3’-diaminobenzidine
